# Global distribution of soapberries (*Sapindus* L.) habitats under current and future climate scenarios

**DOI:** 10.1038/s41598-021-98389-8

**Published:** 2021-10-05

**Authors:** Jiming Liu, Lianchun Wang, Caowen Sun, Benye Xi, Doudou Li, Zhong Chen, Qiuyang He, Xuehuang Weng, Liming Jia

**Affiliations:** 1 Key Laboratory of Silviculture and Conservation of the Ministry of Education, Beijing Forestry University, 35 E Qinghua Rd., Beijing, 100083 People’s Republic of China; 2 National Energy R&D Center for Non-Food Biomass, Beijing Forestry University, Beijing, 100083 People’s Republic of China; 3 College of Forestry, Southwest Forestry University, Kunming Yunnan, 650224 People’s Republic of China; 4 College of Forestry, Nanjing Forestry University, Nanjing, Jiangsu 210037 People’s Republic of China; 5 China Jiliang University, Hangzhou, Zhejiang 310018 People’s Republic of China; 6 Yuanhua Forestry Biological Technology Co., Ltd., Sanming, Fujian 650216 People’s Republic of China

**Keywords:** Ecology, Plant sciences

## Abstract

*Sapindus* (*Sapindus* L.) is a widely distributed economically important tree genus that provides biodiesel, biomedical and biochemical products. However, with climate change, deforestation, and economic development, the diversity of *Sapindus* germplasms may face the risk of destruction. Therefore, utilising historical environmental data and future climate projections from the BCC-CSM2-MR global climate database, we simulated the current and future global distributions of suitable habitats for *Sapindus* using a Maximum Entropy (MaxEnt) model. The estimated ecological thresholds for critical environmental factors were: a minimum temperature of 0–20 °C in the coldest month, soil moisture levels of 40–140 mm, a mean temperature of 2–25 °C in the driest quarter, a mean temperature of 19–28 °C in the wettest quarter, and a soil pH of 5.6–7.6. The total suitable habitat area was 6059.97 × 10^4^ km^2^, which was unevenly distributed across six continents. As greenhouse gas emissions increased over time, the area of suitable habitats contracted in lower latitudes and expanded in higher latitudes. Consequently, surveys and conservation should be prioritised in southern hemisphere areas which are in danger of becoming unsuitable. In contrast, other areas in northern and central America, China, and India can be used for conservation and large-scale cultivation in the future.

## Introduction

*Sapindus* (*Sapindus* L.) is a genus containing 13 evergreen and deciduous tree species in the Sapindaceae family. It is globally distributed across warm-temperate and tropical regions in Southeast Asia and North and South America^1^. *S. saponaria* L. is the type species of the *Sapindus* genus, and it is widely distributed in North and South America, with some localised distributions in Africa and Australia. *S. mukorossi* Gaertn. is the second most widespread species and is found in East and Southeast Asia. *Sapindus* seed oil is high yielding (26.15–44.69%) with a high medium-chain monounsaturated fatty acid content^2^. Crude extracts from the fruit pericarps of *Sapindus* are also rich in triterpenoid saponins (4.14–27.04%) and sesquiterpenoids^3^, such as Saponin A, mukurozi-saponin G, and sapinmusaponin K^4^, which exhibit excellent surface activity as well as antibacterial^5^, elution^6–8^, antibacterial^9,10^, insecticidal^11^, pharmacological^12^, and physiological^13^ effects. Saponin serves as an efficient natural surfactant in commercial soaps, shampoos, and cosmetic cleansers^14^. In recent years, *Sapindus* soap products have been sold in the US, Europe and China, with a market of over 10 million sales in China. Therefore*, Sapindus* species are regarded as economically important sources of biodiesel, as well as biomedical and multi-functional products^1,15^. *Sapindus* germplasm resources are generally scattered in the form of single plants or extremely small populations. With global deforestation and rapid economic development, the diversity of *Sapindus* have been persistently damaged or lost^1^. Modern cultivation of *Sapindus* species has only begun recently and lacks support from relevant research. It is still in a state of low yield with elite varieties lacking and severe germplasm destruction ongoing^1,4^. We suspect that the diversity and habitat of *Sapindus* will suffer further damage in the background of future climate change. Therefore, the protection and management of the core *Sapindus* distribution areas should be strengthened, and natural populations at risk of destruction should be protected through in situ or ex situ conservation efforts.

Niches are habitats with the minimum thresholds necessary for survival^16^. The forest niche is strongly affected by the environment, and it changes or moves with environmental change. The Intergovernmental Panel on Climate Change (IPCC) estimates that a 0.2 °C temperature increase will occur in each future decade that is subject to greenhouse gas emissions^17^. Temperatures will rise by a maximum of 2.6–4.8 °C or a minimum of 0.3–1.7 °C in the twenty-first century^17^. In the face of upcoming rapid climate change, forest trees will unlikely be able to adjust their range with sufficient speed to colonise suitable areas. Species extinction rates would subsequently increase, and warmer temperatures may impact plant growth and yield^18–20^. Hence, there is an urgent need to understand the extent of climate change in the coming decades, and the use of alternative methods to assess its impact on forest tree habitats will be helpful for designing conservation and cultivation plans in the future^21,22^.

Species distribution modelling is an emerging research field based on niche theory. Its principle is to infer the ecological needs of species through mathematical models based on occurrence data and environmental variables, and to create a statistical or mechanistic model of its potential distribution^22–24^. At present, the commonly used niche models are GARP (Genetic Algorithm for Rule-set Production)^25^, MaxEnt (Maximum entropy modeling)^26^, Bioclim^27^, Random Forest^28^, and the Boosted Regression Tree^29^. Many model intercomparison studies have reported that the MaxEnt model, which is based on the principle of maximum entropy^23,26,30^, typically outperforms other species distribution models (SDMs) in terms of high tolerance and high predictive accuracy^31–33^. Over the past 10 years, worldwide research teams have achieved excellent results in the study of rare animal and plant diversity protection^34–38^, invasive species risk prediction^39–41^, marine ecosystem protection^42,43^, disaster distribution prediction^44^, and disease propagation^45,46^ using the MaxEnt model. Zhang et al.^47^ found that the area of suitable range of *Cinnamomum camphora* (L.) Presl will increase and continue to move to the northwest of China under future climate change scenarios. Peng et al.^48^ simulated the distribution of suitable areas for the new oil crop *Paeonia ostii* and found that *Paeonia ostii* will increase its suitable area at high latitudes while decrease at low latitudes. And there were a decreasing tendency observed for three *Coptis* herbal species under the current and future habitat distributions^49^. It is noteworthy that most previous studies have focused on the prediction of suitable habitats in local regions with models that basically follow the default parameters, resulting in suboptimal model accuracy^48,50^. At present, there are no reports or studies, to our knowledge, that focus on current and future suitable habitat projections on a global scale for *Sapindus*.

Based on 5674 global occurrence data records for *Sapindus*, historical environmental factors, and future climate models combined with ArcGIS 10.5 and MaxEnt modelling, we predicted the current global distribution of suitable habitats for *Sapindus* and its response to future climate change scenarios. This study had three objectives: (1) to evaluate the main environmental factors affecting the distribution of *Sapindus*, (2) to explore the distribution of suitable habitats for *Sapindus* under current environmental conditions, and (3) to predict the redistribution pattern of potential *Sapindus* habitats in response to future climate change scenarios in the twenty-first century, and (4) to identify hotspots of habitat degradation and expansion to facilitate climate change-adaptive biological conservation recommendations.

## Results

### Modelling evaluation and selection

We implemented model performance screening using the SDMtoolbox data analysis package in ArcGIS. After simulating regularisation multipliers of 0.5, 1, and 2, combined with five feature type (linear, product, hinge, quadratic, and threshold) model parameters, model performance was assessed based on the AUC values. According to the evaluation results, we found that the MaxEnt model with a regularisation multiplier of 1 and a feature type of LQ (linear and quadratic) parameters was the most successful. Therefore, we applied this parameter model to conduct further analysis. The MaxEnt model for current *Sapindus* suitable habitats provided satisfactory results, with an TSS and AUC value of 0.811 and 0.835, respectively. And The MaxEnt model for future *Sapindus* suitable habitats also achieved satisfactory results, with AUC values ranging from 0.838 to 0.848, and TSS values ranging from 0.810 to 0.821.

### Current suitable habitats for Sapindus

The MaxEnt model's internal jackknife test of factor importance showed that the minimum temperature of the coldest month (Bio6, 26.0% of variation), soil moisture (Sm, 14.1% of variation), mean temperature of the driest quarter (Bio9, 13.1% of variation), mean temperature of the wettest quarter (bio8, 11.6% of variation), and soil pH (Sph, 10.5% of variation) were the major contributors to the *Sapindus* distribution model, with a cumulative contribution of 75.3% (Table 1). Soil carbon, annual mean temperature (Bio 1), annual mean UVb (AmUV) and 13 other indicators contributed 24.7% of the cumulative contribution. According to the MaxEnt results and environmental factor response curves (Fig. S1), the ecological thresholds for the critical environmental factors were the minimum temperature of the coldest month (0–20 °C), soil moisture (40–140 mm), mean temperature of the driest quarter (2–25 °C), mean temperature of the wettest quarter (19–28 °C), and soil pH (5.6–7.6).

**Table 1:** Contributions and thresholds of the major environmental factors in the MaxEnt models for the current suitable habitat of *Sapindus*.

Code	Environmental factor	Percent contribution (%)	Suitable threshold	Units
Bio6	Min temperature of coldest month	26	0–20	°C
Sm	Soil moisture	14.1	40–140	mm
Bio9	Mean temperature of driest quarter	13.1	2–25	°C
Bio8	Mean temperature of wettest quarter	11.6	19–28	°C
Sph	Soil pH	10.5	5.6–7.6	

**Figure 1:**
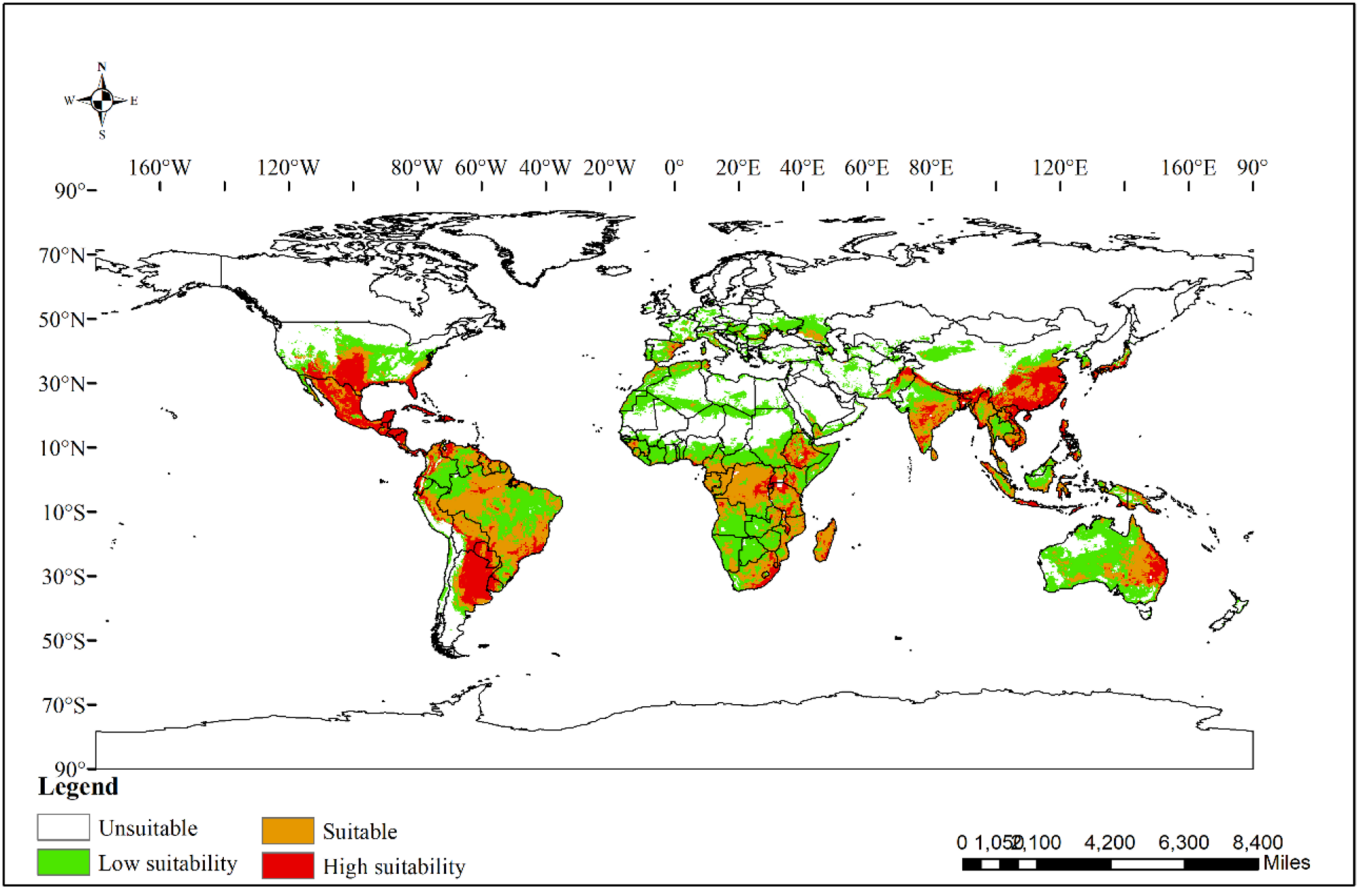
Map of suitable habitat areas for *Sapindus* under current climate conditions. Created in ESRI ArcMap 10.5 (https://support.esri.com/en/Products/Desktop/arcgis-desktop/arcmap).

The suitable habitats for *Sapindus* (Fig. 1) were widely distributed across six continents, except Antarctica, and were mainly distributed in southern and south-eastern Asia, southern North America, northern and central South America, central and southern Africa, and eastern Oceania. The area of total suitable habitat was 6059.97 × 10^4^ km^2^, accounting for 40.67% of the global land area. Among them, low suitability areas accounted for a relatively high area of 2843.00 × 10^4^ km^2^, accounting for 19.08% of the global land area. The suitable and high suitability areas were 2092.92 × 10^4^ km^2^ (14.05%) and 1124.04 × 10^4^ km^2^ (7.54%), respectively (Table 2).

**Table 2:** Area and proportion of different suitability levels for global *Sapindus* habitats under the current environment.

Suitable level	Distribution area (10^4^ km^2^)	Distribution area ratio (%)
Unsuitable	8840.03	59.33
Low suitability	2843.00	19.08
Suitable	2092.92	14.05
High suitability	1124.04	7.54

**Figure 2:**
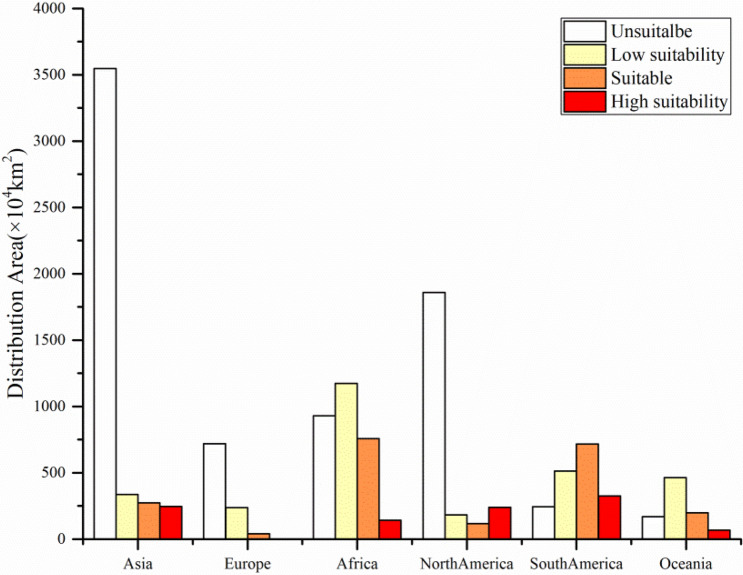
Distribution area of *Sapindus* habitats on six continents under the current environment.

Suitable habitats for *Sapindus* were unevenly distributed across six continents (Fig. 2). The highest area of total suitable habitat was in Africa (2072.258 × 10^4^ km^2^), which contained mainly low suitability areas (1174.139 × 10^4^ km^2^) and a small amount of suitable areas (756.24 × 10^4^ km^2^) and high suitability (141.88 × 10^4^ km^2^) areas. The second highest area was in South America, with a total suitable habitat area of 1,557.337 × 10^4^ km^2^ composed of suitable areas (717.43 × 10^4^ km^2^) and high suitability (326.21 × 10^4^ km^2^) areas. The suitable habitat area for *Sapindus* in Asia was 853.61 × 10^4^ km^2^, of which the suitable and high suitability areas were 272.20 × 10^4^ km^2^ and 245.13 × 10^4^ km^2^, respectively. The suitable habitat area for *Sapindus* in Europe was the lowest of all at only 281.24 × 10^4^ km^2^, of which the suitable and high suitability areas were 41.20 × 10^4^ km^2^ and 2.05 × 10^4^ km^2^, respectively. The high suitability habitats for *Sapindus* were concentrated in south-central South America, including Argentina, Paraguay, and Uruguay; southern North America, including the southern United States, Mexico, and Central American countries; south-eastern Asia, including southern China, northern Vietnam, Bangladesh, northern and central India, Nepal, and northern Pakistan; and very few areas of eastern Oceania and central Africa.

### Potential distribution of *Sapindus* under future climate conditions

**Figure 3:**
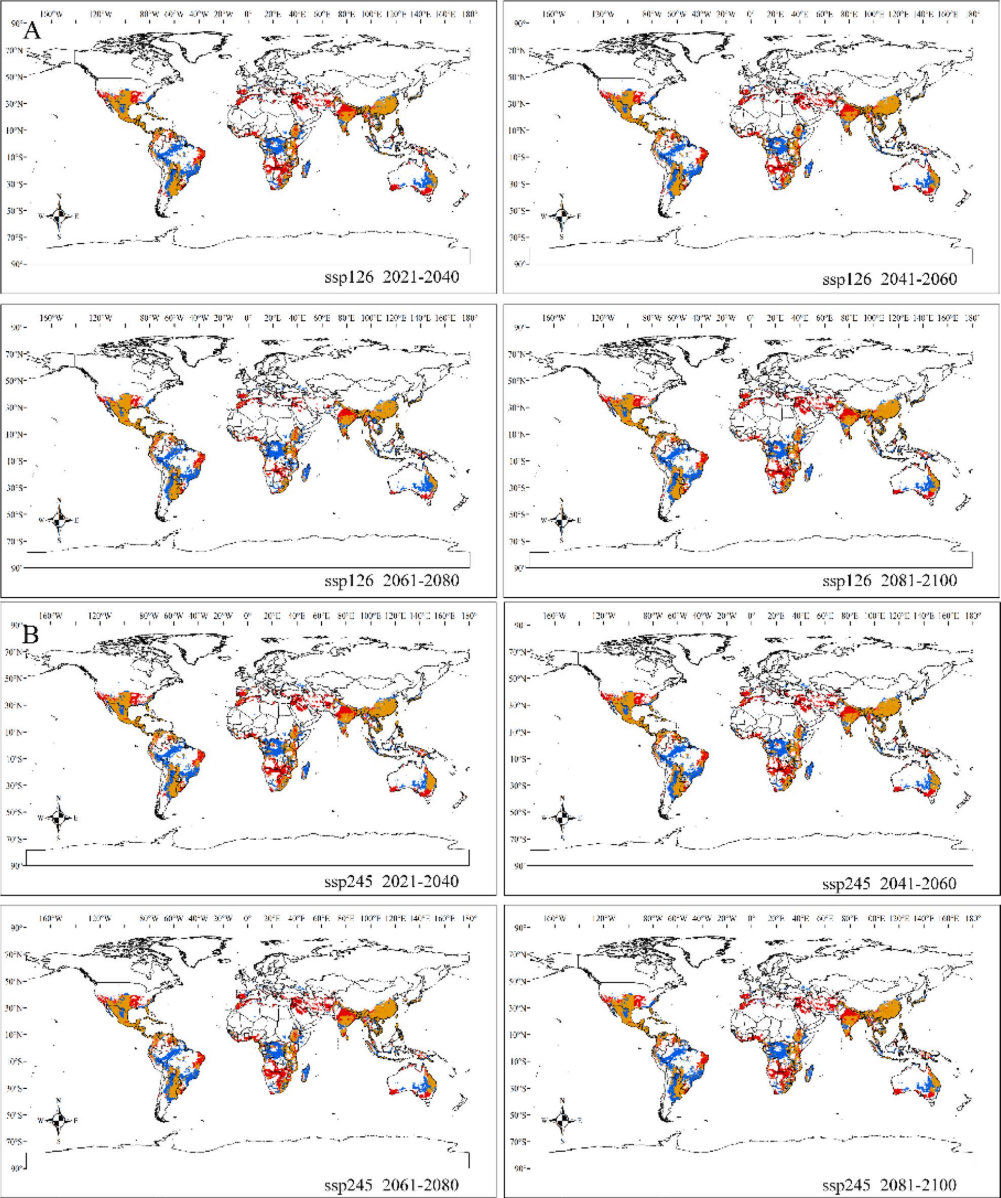

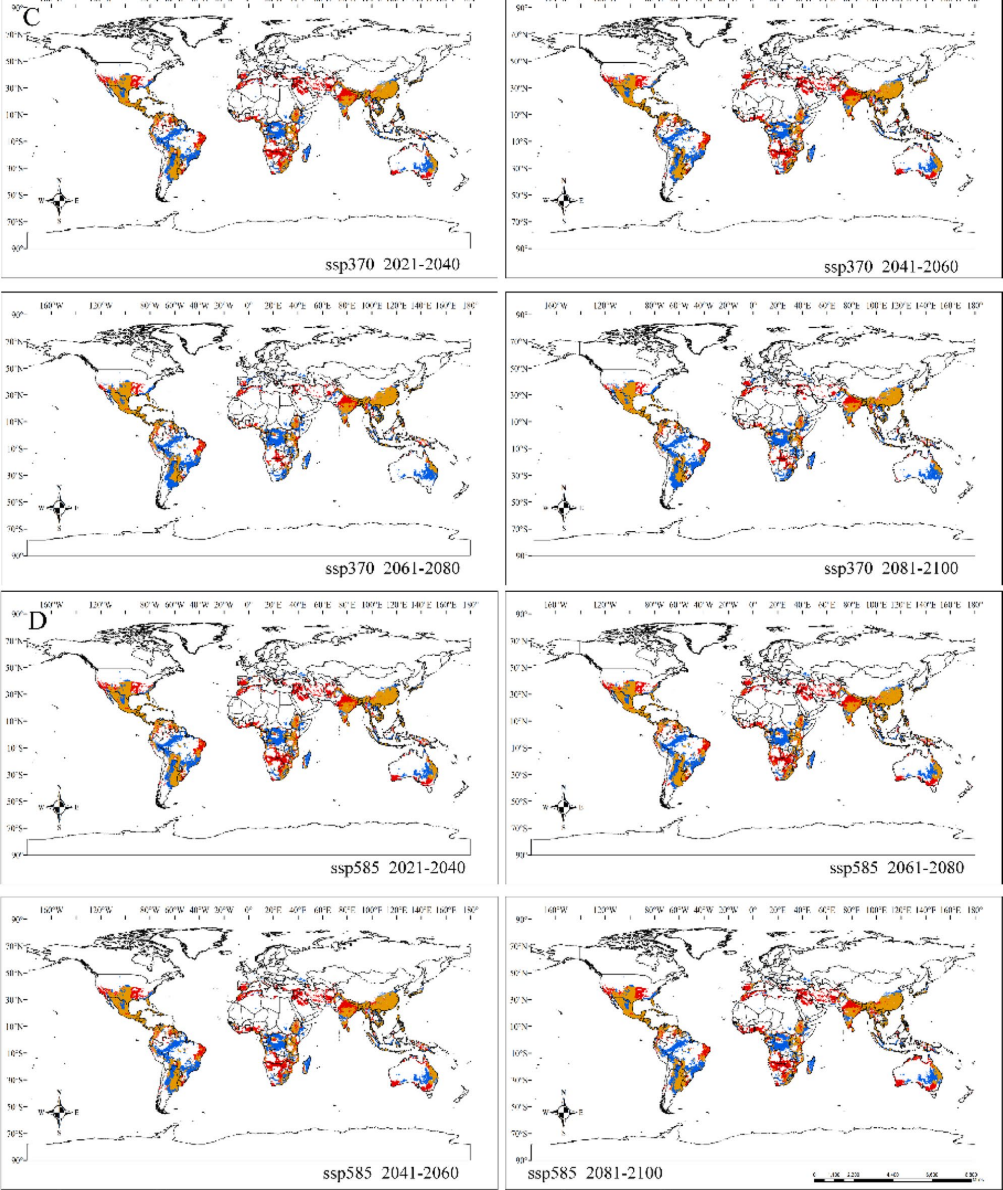
Changes in the distribution of potential suitable habitats for *Sapindus* under the ssp126 (**A**), ssp245 (**B**), ssp370 (**C**), and ssp585 (**D**) scenarios from years 2020 to 2100 compared with the current distribution of suitable habitats. Note: Red areas indicate habitat expansion, yellow areas indicate no change and blue areas indicate habitat contraction. Created in ESRI ArcMap 10.5 (https://support.esri.com/en/Products/Desktop/arcgis-desktop/arcmap).

By comparing the current suitable habitats (Fig. 1) with the projected suitable habitats from 2020 to 2100, we predicted the potential redistribution of *Sapindus* habitats in response to climate change in the twenty-first century under four climate scenarios (Fig. 3). Different trends emerged under the different future climate scenarios. The expansion area of total suitable habitat ranged from 607.45 × 10^4^ km^2^ (ssp370, 2061–2080) to 1092.86 × 10^4^ km^2^ (ssp585, 2081–2100), and the contraction area ranged from 1041.73 × 10^4^ km^2^ (ssp245, 2021–2040) to 1267.23 × 10^4^ km^2^ (ssp370, 2081–2100). Overall, there was a significant contraction in the size of suitable habitats with increasing greenhouse gas emissions. It is noteworthy that the contraction was most pronounced in the second half of the twenty-first century in the southern hemisphere, including South America, Central Africa, and Oceania.

There was significant expansion and contraction of suitable habitats for *Sapindus* in all the future climate scenarios and these expansions and contractions differed significantly between continents (Table S1). Intriguingly, suitable habitats in Asia, Europe, and North America all showed expansion at higher latitudes, and the expansion of Asian habitats ranged from 164.84 × 10^4^ km^2^ (ssp126, 2061–2080) to 293.40 × 10^4^ km^2^ (ssp245, 2061–2080). European habitat expansion ranged from 3.94 × 10^4^ km^2^ (ssp370, 2061–2080) to 32.34 × 10^4^ km^2^ (ssp245, 2020–2040), while expansion of the North American habitats ranged from 79.19 × 10^4^ km^2^ (ssp370, 2061–2080) to 127.39 × 10^4^ km^2^ (ssp245, 2020–2040). The expansion of Asian habitats mainly occurred in northern India, Afghanistan, and the Middle East. The European habitat expansion areas were mainly located in Spain, Portugal, Italy, and Greece, and the North American habitat expansion areas spanned from the southern United States to the central part of the country, including California, Arkansas, Tennessee, and Missouri (Fig. 3).

Conversely, suitable habitats for *Sapindus* in the southern hemisphere contracted significantly, with South American habitat areas shrinking by 405.54 × 10^4^ km^2^ (ssp126, 2041–2060) to 471.38 × 10^4^ km^2^ (ssp370, 2081–2100), which was 2.96 to 3.97 times the expansion area (118.79 × 10^4^ km^2^ [ssp126, 2061–2080] to 136.80 × 10^4^ km^2^ [ssp245, 2020–2040]). Oceania contracted by 94.46 × 10^4^ km^2^ (ssp245, 2020–2040) to 159.70 × 10^4^ km^2^ (ssp370, 2081–2100), which was 0.74 to 15.63 times the expansion area (10.22 × 10^4^ km^2^ [ssp370, 2061–2080] to 126.73 × 10^4^ km^2^ [ssp585, 2081–2100]). African habitat areas contracted by 324.27 × 10^4^ km^2^ (ssp585, 2041–2060) to 402.50 × 10^4^ km^2^ (ssp370, 2081–2100), which was 0.86 to 2.11 times the expansion area (191.19 × 10^4^ [ssp370, 2061–2080] to 378.75 × 10^4^ km^2^ [ssp585, 2081–2100]) (Table S1). The contracted areas were mainly located in Brazil, Peru, Bolivia, Paraguay, and Argentina in South America; the Congo, Mozambique, and Madagascar in Africa; and eastern Australia in Oceania (Fig. 3).

## Discussion

There are 13 species of *Sapindus* worldwide, all of which are scattered in ecosystems as either individual plants or small populations^1^. With global deforestation, rapid expansion of industrial, agricultural, and urban land use, increasing human interference, and over-exploitation of groundwater in the last century, *Sapindus* germplasm resources have been persistently damaged or lost^3^. Therefore, it is imperative to sustainably exploit and conserve *Sapindus* germplasm diversity by predicting the distribution of current and future suitable habitats through ecological niche modelling.

This study analysed projections of current and future distributions of suitable habitats for *Sapindus* using the MaxEnt model. Habitat maps were created for *Sapindus* based on occurrence data sets with great TSS and AUC valuesfor the present and the future, respectively. Therefore, we consider that our model performance is robust and adequate for construing the overall suitable habitat distribution of *Sapindus*. To our knowledge, this is the first study to analyse the suitable habitat distribution of *Sapindus* for the current and future using the MaxEnt model.

### Suitable habitat distribution patterns of *Sapindus* under the current environment

After a long period of natural selection, anthropogenic disturbance, and geographic isolation, genetic variation in the *Sapindus* genus has increased greatly, resulting in patterns of geographic variation in both phenotype and ecological adaptation^3,51,52^. According to the response curves of ecological factors, the relationships between the probability of species occurrence and the main ecological factors can be determined, and it is generally considered that if the probability of species occurrence is greater than 60%, the corresponding ecological factor thresholds are suitable for the survival of this species^30,53,54^. In our study, the MaxEnt results and environmental factor response curves indicated that the critical environmental factors affecting suitable habitats for *Sapindus* were minimum temperature of coldest month, soil moisture, mean temperature of driest quarter, mean temperature of wettest quarter, and soil pH. Similarly, Adeyemi found that the minimum temperature of the coldest month was the most critical environmental factor in determining the suitable habitat distribution of Sapindaceae Juss. species in West Africa^55^. Sun et al.^56,57^ also found that the annual minimum temperature played an important role in *Sapindus* saponin variation, but high precipitation levels were found to inhibit saponin synthesis^55–57^. Liu found that the precipitation of the warmest quarter and isothermality were the critical environmental factors that determine the distribution of *Sapindus* habitats in China. *Sapindus* is highly thermophilous^3,58,59^ and xerophilous tree species^1^ that prefer areas with abundant heat and sunlight resources^3^. They do not tolerate excessive cold and humidity. Moderate rainfall and soil conditions are more conducive to their survival^60^. On a global scale, we found that the critical environmental factor for *Sapindus* survival was the minimum temperature of the coldest month. However, extremely low temperatures (below -10 ℃) are also detrimental to the survival of *Sapindus*^61^. This indicates that the current suitable habitats for *Sapindus* are likely to be mainly distributed in tropical and subtropical regions between the 50° north parallel and the 40° south parallel latitudes. This is consistent with the findings of Liu^60^, who found that the northernmost margin of *Sapindus* distribution in China did not go beyond the Qinling Mountains or the Huai River (for the most part). *Sapindus* is deep-rooted and drought-tolerant^62^ tree species that does not require high levels of soil moisture^63^.We have found that soils with a soil moisture ranges of 40 to 140 mm and weak acidity or neutrality (soil Ph 5.6–7.6) are suitable for the survival of *Sapindus*.

Our results indicated that the total suitable habitat area for *Sapindus* globally was 6059.97 × 10^4^ km^2^. Of this, the proportion of low suitability areas accounted for 46.91%, suitable areas accounted for 34.54%, and high suitability areas accounted for 18.55%. The total suitable habitats for *Sapindus* were found to be unevenly distributed across six continents, with high suitability areas concentrated in the Mississippi Plain, the Florida Peninsula, and Mexico in North America; La Plata Plain in South America; and the North China Plain, Middle and Lower Yangtze Plain, Sichuan Basin, Yunnan-Guizhou Plateau, and Southern Himalayas in Asia. These results are consistent with a previous study, which found that *Sapindus* are mainly distributed in America and Asia^1,64^. High suitability areas are more conducive to cultivation and the conservation of *Sapindus* diversity than low suitability and suitable habitats. Therefore, it is recommended that ex situ conservation, cultivation, and breeding of *Sapindus* species be implemented in high-suitability areas.

### Response of suitable habitats for *Sapindus* to future climate change

Climate plays a significant role in defining species distributions, and changes in the distribution of species are also the most clear and direct response to climate. Global warming may substantially change the structure and function of terrestrial ecosystems, resulting in significant changes in the extent and distribution of biological habitats^16,20^.

Our results indicated that the size and distribution of suitable habitats for *Sapindus* varied under different climatic scenarios, suggesting that climate change had an uncertain and region-specific effect on the distribution of these habitats. Under future climate scenarios, we estimate that suitable habitats for *Sapindus* will expand and contract significantly across continents. These habitats will significantly contract in lower latitudes and expand in higher latitudes. Our model showed that as greenhouse gas emissions increased and time passed, the area of suitable habitats contracted sharply, peaking in the second half of the twenty-first century (2081–2100). In terms of the northern and southern hemispheres, the *Sapindus* suitable habitats for *Sapindus* in the northern hemisphere predominantly expanded, while in the southern hemisphere they predominantly contracted. Jayasinghe and Prevéy also found a declining trend in the area of suitable habitats for *Camellia sinensis* (L.) O. Kuntze^65^ and huckleberry^54^ in the face of global warming, while He^66^ found an expanding trend in the highly suitable habitat area for *Xanthoceras sorbifolia* Bunge under global warming in China. The *Sapindus* genus consists of drought-tolerant and hardy species, but they are more sensitive to extreme temperatures. With future global warming, parts of the high latitudes of the northern and southern hemispheres may be transformed into suitable habitats for *Sapindus* due to an increase in extreme low temperatures, while parts of the lower latitudes may experience persistent extreme heat or drought as the climate continues to warm. This poses a major problem for *Sapindus* survival. In He’s study^66^, the distribution of *X. sorbifolia* was observed to be localised in areas of the Loess Plateau in China, which is at a higher latitude. Thus, a trend toward the expansion of suitable cultivation areas in the face of global warming is already evident. Our results indicated that *Sapindus* will also experience some localised suitable habitat expansions in the northern hemisphere at higher latitudes, but, generally, *Sapindus* habitats will predominantly contract in the face of global warming. The southern hemisphere, including the Congo Basin and Madagascar Island in Africa; the Amazon Plains, the Brazilian Plateau, and La Plata Plain in South America; and the central plains of Oceania, may no longer be suitable for *Sapindus* in the face of future global warming. These areas should be prioritised for surveys and collection of *Sapindus* germplasms^3,15,56^. Some elite or distinctive germplasms could be ex situ-conserved by vegetative propagation in order to conserve as much *Sapindus* genetic diversity as possible. The Mississippi Plain, the Florida Peninsula, Mexico, Central America, the Middle and Lower Yangtze Plain, the Yungui Plateau, and central India, which are relatively less affected by climate change, can be used as a base for resource conservation, large-scale cultivation, and utilisation of *Sapindus* in the future.

Although the MaxEnt model is the most commonly used model in recent years for predicting species distribution change and has greater prediction performance than other species distribution prediction models, it still has some limitations^53^. Despite climatic, soil, topographical*,* and solar radiation factors being taken into account in this model, species distributions can also be constrained by other factors such as adaptive capacity, interspecific interactions, human activities, land use. When all factors are considered together, we estimate that the area of suitable habitats for *Sapindus* will further reduce and the contraction in habitat area in the face of future global warming may be more significant. However, importing all variables into the model may lead to difficulties in variable screening, the effects of key variables may be weakened, and the resulting simulations may not necessarily be more accurate than the current model. Therefore, our findings on the distribution pattern of suitable habitats for *Sapindus* and its response to future climate change, based on the MaxEnt model, provide an important theoretical basis and valuable recommendations for the conservation and sustainable exploitation of *Sapindus* genetic diversity.

## Conclusions

Our modelling study showed that temperature, soil moisture, and soil pH may play an important role in determining the global distribution of suitable habitats for *Sapindus*. The ecological thresholds for critical environmental factors affecting *Sapindus* were the minimum temperature of the coldest month (0–20 °C), soil moisture (40–140 mm), mean temperature of the driest quarter (2–25 °C), mean temperature of the wettest quarter (19–28 °C), and soil pH (5.6–7.6). We found that the total suitable habitat area for *Sapindus* was 6059.97 × 10^4^ km^2^, which was unevenly distributed across six continents. High suitability areas were found to be concentrated in the Mississippi Plain, Florida Peninsula, and Mexico in North America; La Plata Plain in South America; and the North China Plain, Middle and Lower Yangtze Plain, Sichuan Basin, Yunnan-Guizhou Plateau, and Southern Himalayas in Asia. By integrating simulations from four future climate scenarios from 2020 to 2100, the size and distribution of suitable habitats for *Sapindus* were found to vary significantly across different continents depending on the climatic scenario. As greenhouse gas emissions increased (SSP126 to SSP585) and time passed (2021 to 2100), the overall area of suitable habitats contracted to an increasing degree, resulting from a significant contraction in lower latitudes and a slight expansion to higher latitudes. Suitable habitats for *Sapindus* in the northern hemisphere predominantly expanded, while in the southern hemisphere they contracted under the future climate change. Therefore, germplasm surveys and resource conservation should be prioritised in areas of the southern hemisphere that will likely become unsuitable for *Sapindus* in the future. In contrast, the Mississippi Plain, the Florida Peninsula, Mexico, Central America, the Middle and Lower Yangtze Plain, the Yungui Plateau, and central India can be used as a base for resource conservation, large-scale cultivation, and utilisation of *Sapindus* in the future.

## Materials and methods

### Design framework

**Figure 4:**
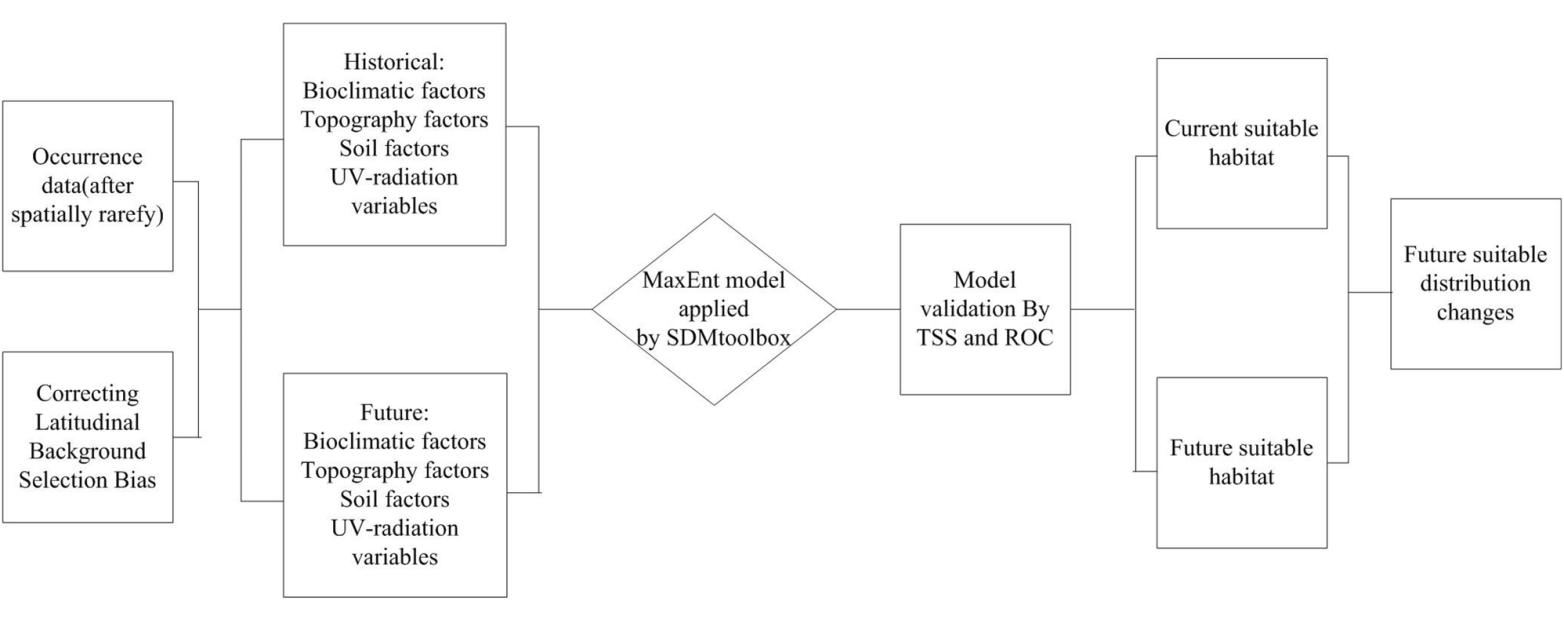
Design framework of the suitable habitat distribution model for *Sapindus*.

Based on global *Sapindus* occurrence data combined with historical environmental factors and future climate models, we explored the current and future potential habitats of *Sapindus* by utilising the MaxEnt (MaxEnt version 3.4.1) model and ArcGIS (ArcGIS 10.5). The specific process is shown in Fig. 4.

### *Sapindus* occurrence records

**Figure 5:**
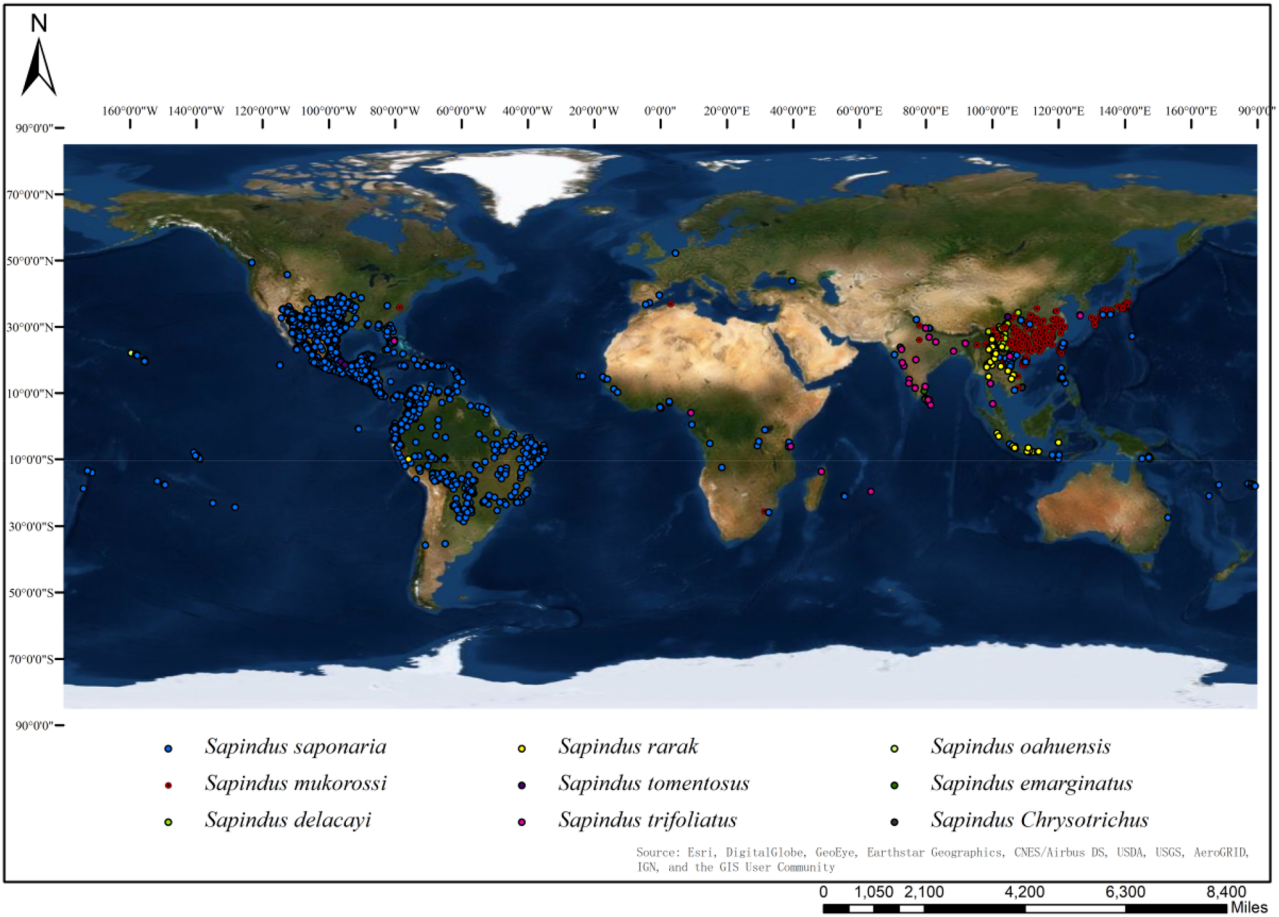
Global distribution of occurrence records for *Sapindus*. Created in ESRI ArcMap 10.5 (https://support.esri.com/en/Products/Desktop/arcgis-desktop/arcmap).

We collected 5674 worldwide *Sapindus* occurrence records from well-designed surveys, including: (1) 205 occurrence records from field germplasm surveys of *Sapindus* in China^3^, (2) 21 occurrence records from the Chinese National Plant Specimen Resource Center (CVH, http://www.cvh.ac.cn/), (3) 5425 occurrence records from the Global Biodiversity Information Facility (GBIF, https://www.gbif.org/)^67^, and (4) 23 occurrence records from the Chinese National Specimen Information Infrastructure (NSII, http://www.nsii.org.cn/2017/). There were 4505 *S. saponaria*, 893 *S. mukorossi*, 108 *S. delavayi* (Franch.) Radlk., 84 *S. rarak* DC., 50 *S. trifoliatus* L., 15 *S. oahuensis*, 12 *S. emarginatus* Vahl, 4 *S. tomentosus* Kurz, and 3 *S. chrysotrichus* germplasms. However, there were some spatial clusters of occurrence records, especially in southern North America and Southeast Asia. When these spatial clusters exist, models are often over-fitted in terms of environmental biases, and model performance values are inflated^68,69^. Therefore, we employed the Spatially Rarefy Occurrence Data tool in SDMtoolbox 2.0^70^ and set the spatial interval at 10 km to eliminate spatial clusters of occurrences. After elimination, 2245 occurrence records were used in the model (Fig. 5).

### Environmental parameters

There were 32 environmental factors used in this projection, including bioclimatic, topographical, UV-radiation, and soil parameters, to model the potential suitable habitat for *Sapindus* (Table S2). The bioclimatic factors for current climate conditions were extracted from the 2.5-min resolution historical climate (average for 1970–2000) database (https://www.worldclim.org/data/worldclim21.html). Topographical factors were obtained from the Harmonized World Soil Database v1.2 from the Food and Agriculture Organization of the United Nations (http://www.fao.org/soils-portal/soil-survey/soil-maps-and-databases/). Soil factors were extracted from the Center for Sustainability and the Global Environment dataset (https://nelson.wisc.edu/sage/). UV-radiation variables were obtained from the glUV dataset (https://www.ufz.de/gluv/)^71^.

Future climate projections were extracted from the 2.5-min resolution Shared Socioeconomic Pathways (SSPs) scenarios available from the BCC-CSM2-MR global climate (2021–2040, 2041–2060, 2061–2080, and 2081–2100) database of the Coupled Model Intercomparison Projects 6 (CMIP6) (https://www.worldclim.org/), which is a project of the World Climate Research Programme (WCRP)’s Working Group of Coupled Modelling (WGCM). This included the SSP1-2.6 (ssp126), SSP2-4.5 (ssp245), SSP3-7.0 (ssp370), and SSP5-8.5 (ssp585) scenarios, all of which were considered possible future climates depending on the quantity of greenhouse gases emitted in the near future. All environmental factors were statistically resampled to a 2.5-min resolution using ArcGIS.

However, there were several environmental factors that may have exhibited collinearity. Strong collinearity between these factors may artificially inflate the accuracy of a model^72^. In this study, we applied the remove highly correlated variables tool in SDMtoolbox to avoid the potential problem of multicollinearity among environmental factors. Set 0.9 as the maximum correlation allowed value, we retained 21 environmental factors for subsequent modelling, respectively were Annual mean temperature (Bio1), Mean diurnal range (Bio2), Isothermality (Bio3), Max temperature of warmest month (Bio5), Min temperature of coldest month (Bio6), Temperature annual range (Bio7), Mean temperature of wettest quarter (Bio8), Mean temperature of driest quarter (Bio9), Mean temperature of warmest quarter (Bio10), Annual precipitation (Bio12), Precipitation seasonality (Bio15), Precipitation of wettest quarter (Bio16), Precipitation of driest quarter (Bio17), Precipitation of warmest quarter (Bio18), Precipitation of coldest quarter (Bio19), elevation (Elv), Soil Moisture (Sm), Soil Organic Carbon (Soc), Soil pH (Sph), Annual mean UVb (AmUV), Seasonality UVb (SeaUV).

### Model application

The MaxEnt model utilises the maximum entropy principle, applying five different feature constraints (linear, product, hinge, quadratic, and threshold) to environmental variables to calculate the potential geographic distribution probability of a species^53,73^. We used the SDMtoolbox to run the MaxEnt model on the ArcGIS platform. We set 25% of the occurrence data as testing data and 75% of the occurrence data as training data, output format was cloglog, and applied percentile training presence as threshold rule.To determine the key environmental factors that drive *Sapindus* habitat distribution, we performed a jackknife permutation to rank the environmental factors and visualised the results in response curves. To calibrate and validate the robustness of the MaxEnt model, ture skill statistic (TSS)^74^ and receiver operating characteristic curve (ROC curve) analysis were used, TSS can range from − 1 to 1 and the area under the receiver operating curve (AUC) ranges from 0 to 1^75,76^. Model performance was classified as failing (0.5–0.6), poor (0.6–0.7), fair (0.7–0.8), good (0.8–0.9), and excellent (0.9–1) according to the AUC value^77^. TSS values closer to 1 indicated more successful models^74^.

We converted the continuous suitability score (0–1) of the MaxEnt model output into a habitat distribution visualisation using ArcGIS. We reclassified suitability into four classes: unsuitable habitat (< 0.25), low suitability (0.25–0.50), suitable habitat (0.50–0.75), and high suitability (> 0.75)^78,79^.

In the future distribution model, the future climate scenarios of four greenhouse gas emission models were used to simulate the global migration and change of suitable habitats for *Sapindus* from 2020 to 2100. Topographic and soil factors were set as stabilising variables in future models since topographic and soil factors are largely unaffected by climate change. In order to visualise the expansion and contraction of suitable habitats for *Sapindus* under different future climate scenarios, we created habitat suitability maps using ArcGIS and compared the current suitable habitats with those in each future climate scenario.

## Supplementary Information

Supplementary Information.

## Data Availability

The datasets generated during and/or analysed during the current study are available in the Chinese National Plant Specimen Resource Center (CVH, http://www.cvh.ac.cn/), Global Biodiversity Information Facility (GBIF, https://www.gbif.org/), Chinese National Specimen Information Infrastructure (NSII, http://www.nsii.org.cn/2017/), WorldClim dataset (https://www.worldclim.org/data/worldclim21.html), Harmonized World Soil Database v1.2 from the Food and Agriculture Organization of the United Nations (http://www.fao.org/soils-portal/soil-survey/soil-maps-and-databases/), Center for Sustainability and the Global Environment dataset (https://nelson.wisc.edu/sage/), and glUV dataset (https://www.ufz.de/gluv/).
