# Supplementary material for: Global distribution of soapberries (Sapindus L.) habitats under current and future climate scenarios
Source: Sci Rep. 2021 Oct 5;11:19740. doi: 10.1038/s41598-021-98389-8 (PMC8492679; doi:10.1038/s41598-021-98389-8)
Supplement: Supplementary file 1 — Supplementary Information. [file 41598_2021_98389_MOESM1_ESM.docx]

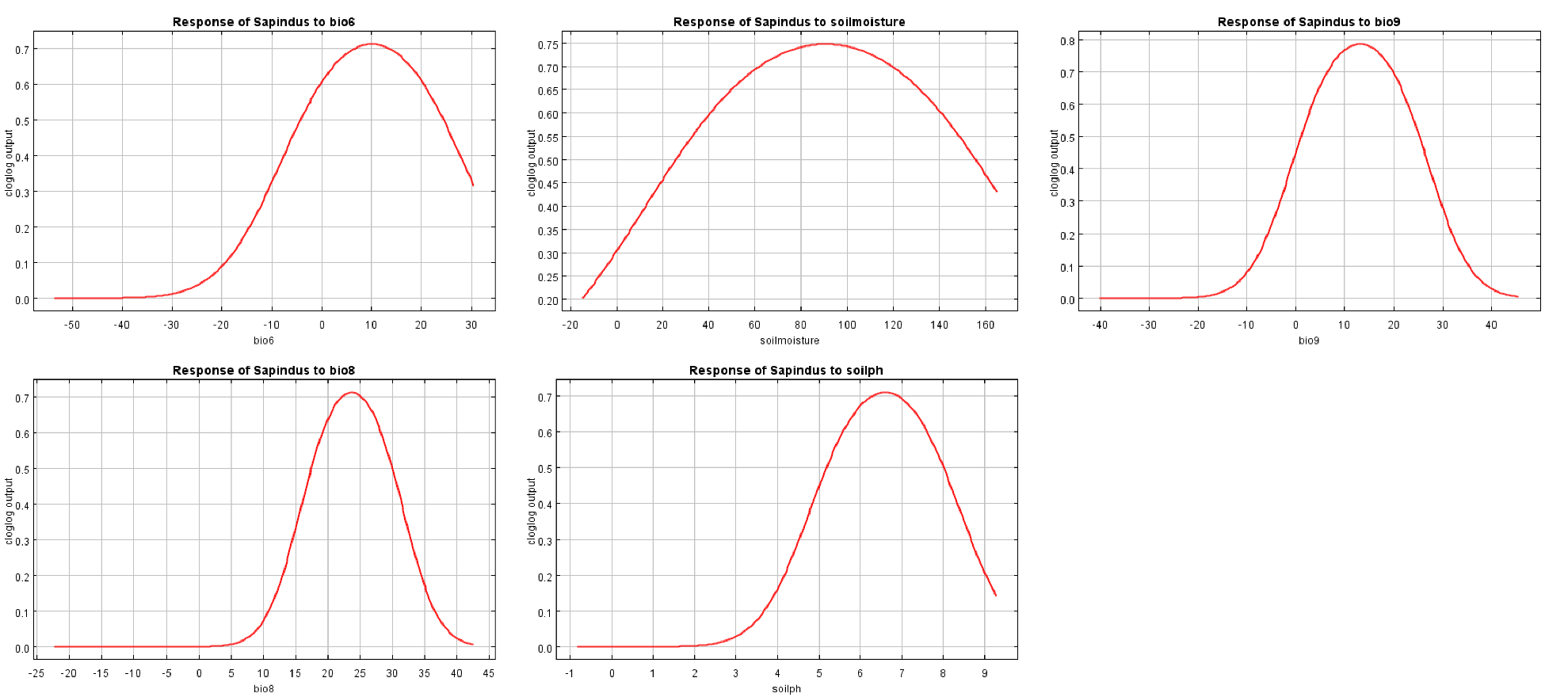


Figure S1. Response curves of *Sapindus* MaxEnt modeling under current climate conditions.

**Table S1** The area change of suitable habitat of *Sapindus* under different future climate scenarios on different continents.

| **Continent** |  | **ssp126** | | | |  | **ssp245** | | | |  | **ssp370** | | | |  | **ssp585** | | | |
| --- | --- | --- | --- | --- | --- | --- | --- | --- | --- | --- | --- | --- | --- | --- | --- | --- | --- | --- | --- | --- |
|  |  | **2020-2040** | **2041-2060** | **2061-2080** | **2081-2100** |  | **2020-2040** | **2041-2060** | **2061-2080** | **2081-2100** |  | **2020-2040** | **2041-2060** | **2061-2080** | **2081-2100** |  | **2020-2040** | **2041-2060** | **2061-2080** | **2081-2100** |
| Asia | Expansion | 267.54 | 269.56 | 164.84 | 277.24 |  | 265.51 | 276.24 | 293.40 | 277.73 |  | 288.51 | 291.69 | 184.88 | 178.77 |  | 286.30 | 278.42 | 281.38 | 283.46 |
|  | Unchange | 569.16 | 570.14 | 547.17 | 569.69 |  | 558.92 | 570.32 | 575.23 | 579.92 |  | 560.71 | 570.39 | 551.21 | 542.96 |  | 563.09 | 568.73 | 567.83 | 571.71 |
|  | Contraction | 174.92 | 173.94 | 196.90 | 174.39 |  | 185.15 | 173.75 | 168.85 | 164.16 |  | 183.37 | 173.69 | 192.86 | 201.12 |  | 180.99 | 175.35 | 176.25 | 172.37 |
|  |  |  |  |  |  |  |  |  |  |  |  |  |  |  |  |  |  |  |  |  |
| Europe | Expansion | 27.56 | 24.51 | 17.55 | 28.99 |  | 32.34 | 29.67 | 25.22 | 21.47 |  | 23.74 | 29.05 | 3.94 | 11.68 |  | 31.41 | 32.18 | 29.24 | 27.75 |
|  | Unchange | 9.16 | 9.29 | 5.33 | 10.11 |  | 10.56 | 9.53 | 8.58 | 8.13 |  | 7.62 | 9.77 | 0.90 | 2.51 |  | 9.98 | 10.51 | 9.49 | 9.73 |
|  | Contraction | 16.11 | 15.99 | 19.95 | 15.17 |  | 14.72 | 15.75 | 16.69 | 17.15 |  | 17.66 | 15.51 | 24.38 | 22.77 |  | 15.30 | 14.77 | 15.79 | 15.55 |
|  |  |  |  |  |  |  |  |  |  |  |  |  |  |  |  |  |  |  |  |  |
| Africa | Expansion | 319.07 | 322.31 | 223.97 | 323.00 |  | 331.28 | 321.22 | 352.16 | 329.45 |  | 328.69 | 340.75 | 191.11 | 211.47 |  | 337.49 | 338.10 | 359.15 | 378.75 |
|  | Unchange | 319.40 | 318.21 | 281.02 | 318.84 |  | 328.75 | 325.80 | 327.79 | 316.31 |  | 316.54 | 323.88 | 251.34 | 249.69 |  | 318.18 | 327.92 | 313.07 | 310.40 |
|  | Contraction | 332.79 | 333.98 | 371.17 | 333.35 |  | 323.44 | 326.39 | 324.40 | 335.88 |  | 335.65 | 328.31 | 400.85 | 402.50 |  | 334.01 | 324.27 | 339.12 | 341.79 |
|  |  |  |  |  |  |  |  |  |  |  |  |  |  |  |  |  |  |  |  |  |
| North America | Expansion | 117.39 | 111.42 | 90.55 | 120.59 |  | 127.39 | 128.12 | 119.96 | 114.93 |  | 120.51 | 122.43 | 79.19 | 76.87 |  | 119.58 | 118.40 | 122.19 | 124.51 |
|  | Unchange | 398.53 | 394.27 | 402.48 | 400.38 |  | 402.36 | 405.31 | 402.33 | 397.28 |  | 396.85 | 401.34 | 393.69 | 393.71 |  | 399.16 | 404.46 | 399.30 | 399.94 |
|  | Contraction | 86.11 | 90.36 | 82.15 | 84.25 |  | 82.27 | 79.32 | 82.30 | 87.35 |  | 87.78 | 83.30 | 90.94 | 90.93 |  | 85.47 | 80.17 | 85.34 | 84.69 |
|  |  |  |  |  |  |  |  |  |  |  |  |  |  |  |  |  |  |  |  |  |
| South America | Expansion | 131.78 | 133.80 | 118.79 | 127.99 |  | 136.80 | 126.74 | 133.61 | 120.73 |  | 136.70 | 136.88 | 113.93 | 109.28 |  | 135.22 | 132.11 | 132.83 | 134.99 |
|  | Unchange | 387.67 | 389.11 | 370.80 | 388.69 |  | 387.28 | 372.13 | 377.78 | 351.34 |  | 384.52 | 383.38 | 330.25 | 323.27 |  | 386.08 | 384.55 | 378.18 | 373.63 |
|  | Contraction | 406.97 | 405.54 | 423.84 | 405.95 |  | 407.36 | 422.51 | 416.86 | 443.30 |  | 410.13 | 411.26 | 464.40 | 471.38 |  | 408.56 | 410.09 | 416.46 | 421.02 |
|  |  |  |  |  |  |  |  |  |  |  |  |  |  |  |  |  |  |  |  |  |
| Oceania | Expansion | 100.91 | 88.81 | 53.56 | 87.78 |  | 93.95 | 85.25 | 93.42 | 86.81 |  | 83.19 | 91.25 | 10.22 | 12.39 |  | 85.03 | 87.65 | 108.47 | 126.73 |
|  | Unchange | 84.96 | 92.26 | 66.79 | 92.08 |  | 115.06 | 101.89 | 98.49 | 93.21 |  | 78.50 | 83.44 | 50.82 | 49.83 |  | 86.85 | 93.25 | 89.28 | 74.26 |
|  | Contraction | 124.57 | 117.26 | 142.73 | 117.44 |  | 94.46 | 107.63 | 111.04 | 116.31 |  | 131.03 | 126.08 | 158.70 | 159.70 |  | 122.67 | 116.28 | 120.24 | 135.27 |

**Table S2.** Environmental factors used in this study and their corresponding codes.

| **Data type** | **Code** | **Environmental factor** | **Units** |
| --- | --- | --- | --- |
| Bioclimatic factor | Bio1 | Annual mean temperature | ×10 ℃ |
|  | Bio2 | Mean diurnal range | ×10 ℃ |
|  | Bio3 | Isothermality | % |
|  | Bio4 | Temperature seasonality |  |
|  | Bio5 | Max temperature of warmest month | ×10 ℃ |
|  | Bio6 | Min temperature of coldest month | ×10 ℃ |
|  | Bio7 | Temperature annual range | ×10 ℃ |
|  | Bio8 | Mean temperature of wettest quarter | ×10 ℃ |
|  | Bio9 | Mean temperature of driest quarter | ×10 ℃ |
|  | Bio10 | Mean temperature of warmest quarter | ×10 ℃ |
|  | Bio11 | Mean temperature of coldest quarter | ×10 ℃ |
|  | Bio12 | Annual precipitation | mm |
|  | Bio13 | Precipitation of wettest month | mm |
|  | Bio14 | Precipitation of driest month | mm |
|  | Bio15 | Precipitation seasonality | % |
|  | Bio16 | Precipitation of wettest quarter | mm |
|  | Bio17 | Precipitation of driest quarter | mm |
|  | Bio18 | Precipitation of warmest quarter | mm |
|  | Bio19 | Precipitation of coldest quarter | mm |
|  | Eva | Evapotranspiration | mm |
|  | Gdd | Growing Degree Days | ℃ |
|  | Sd | Snow Depth | mm |
| Topography factor | Elv | Elevation | m |
| Soil factor | Npp | Net Primary Productivity | kg/m^2^ |
|  | Sm | Soil Moisture | mm |
|  | Soc | Soil Organic Carbon | Kg^-c^/m^2^ |
|  | Sph | Soil pH |  |
|  | Ar | Annual Runoff | mm/yr |
| UV-radiation variables | AmUV | Annual mean UVb | J/m^2^/day |
|  | SeaUV | Seasonality UVb |  |
|  | HighUV | Highest UVb | J/m^2^/day |
|  | LowUV | Lowest UVb | J/m^2^/day |
